# Causal association between genetically predicted circulating immune cell counts and frailty: a two-sample Mendelian randomization study

**DOI:** 10.3389/fimmu.2024.1336498

**Published:** 2024-01-23

**Authors:** Xiao-Guang Guo, Ya-Juan Zhang, Ya-Xin Lu, Jia-Mei Lu, Jie Zhang, Hui-Xin Li, Chao-Jin Chen, Jian-Jun Yang

**Affiliations:** ^1^ Department of Anesthesiology, Pain and Perioperative Medicine, The First Affiliated Hospital of Zhengzhou University, Zhengzhou, China; ^2^ Department of Obstetrics and Gynecology, The First Affiliated Hospital of Zhengzhou University, Zhengzhou, China; ^3^ Big Data and Artificial Intelligence Center, The Third Affiliated Hospital of Sun Yat-sen University, Guangzhou, China; ^4^ Department of Anesthesiology, The Third Affiliated Hospital of Sun Yat-sen University, Guangzhou, China

**Keywords:** immune cell, inflammation, frailty index, Mendelian randomization, eosinophil count

## Abstract

**Background:**

Despite the recognized link between immune responses and frailty, the association between immune cell counts and frailty based on previous observational studies remains disputed, with uncertain causal nexus. This study aimed to elucidate causal association between genetically predicted circulating immune cell counts and frailty.

**Methods:**

We conducted the two-sample Mendelian randomization (MR) study with independent genetic variants associated with six immune cell subtype counts from genome-wide association studies in 563,946 European individuals. Frailty summary data, assessed via frailty index (FI), was obtained from study comprising 175,226 subjects. Univariate MR, reverse MR and multivariate MR were conducted to comprehensive investigate the association between immune cell counts and FI, with two-step MR analysis for mediation analysis.

**Results:**

Univariate MR evidence indicated that among six leukocyte subtype counts, only elevated eosinophil count was significantly correlated with higher FI (β = 0.059, 95% confidence interval [CI], 0.042–0.078, *P*=5.63E-11), with no reverse causal relationship identified in reverse MR. In multivariate MR, the causal effect of eosinophil count retained statistical significance (β = 0.063, 95% CI, 0.021–0.104, *P* = 0.003). Ultimately, the two-step MR analysis demonstrated two mediators in this causal pathway: asthma (β= 0.019, 95% CI, 0.013–0.025, *P* = 35.84E-10, mediated proportion, 31.732%) and rheumatoid arthritis (β= 0.004, 95% CI, 0.001–0.006, *P*=1.75E-03, mediated proportion, 6.411%).

**Conclusions:**

Within immune cell subtypes, MR evidence indicated only genetically predicted circulating eosinophil count had irreversible and independent causal effect on frailty, with asthma and rheumatoid arthritis possibly serving as partial mediators. The finding stressed the need for further exploring physiological functions of eosinophils in order to develop effective strategies against frailty.

## 1 Introduction

Frailty, a multisystem aging syndrome, represents a precarious state defined by a decline in the body’s adaptive capacity and stress resistance when facing stressors, which stems from the cumulative deterioration of multiple systems’ functions over the lifespan (1). Multiple approaches exist for frailty evaluation, but the commonly utilized is frailty index (FI), which employs a cumulative deficit model that integrates multiple physiological and psychological parameters to quantify frailty (2, 3). Increased FI is associated with various adverse outcomes, including additional complications, restricted mobility, prolonged hospital stays and unplanned discharge, and even increased mortality risk (4–7). Although physiological imbalances in multiple systems contribute to frailty, chronic inflammation or worsened immune conditions significantly influence frailty onset (8).

Circulating immune cell and its subsets constitute the primary cellular elements of immune-inflammatory system. Different immune cell subtypes play distinct roles in inflammation and immune response. Recent findings from observational research revealed that systemic inflammation played a pivotal role in identifying high-risk individuals with early-onset frailty, while increased C-reactive protein level and leukocyte count were associated with frailty progression (9). An additional cohort study demonstrated that among different leukocyte subtypes, only neutrophil count exhibited a positive correlation with frailty, whereas lymphocyte count was inversely related to frailty (10). Research focused on elderly women revealed that increased neutrophil and monocyte counts among leukocyte subtypes were significantly associated with frailty (11). Conversely, several studies have indicated non-significant relationship between frailty and leukocyte count or lymphocyte subpopulations (12, 13). Aforementioned controversial findings may have been influenced by population heterogeneity and discrepancies in frailty assessment methods. Importantly, observational research may be compromised by potential biases arising from reverse causation and unmeasured confounders. To date, the causal nexus between circulating immune cell counts and frailty remains indeterminate.

Mendelian randomization (MR) enables estimation of causality between exposure and outcome by utilizing genetic variants as unbiased instrumental variables, while minimizing the impact of potential confounders and reverse causation (14). Additionally, the two-step MR for testing mediators in causal pathways was recently developed, which is less biased than the multivariable method (15). Previous MR research indicated a causal association between immune cell subtype count and the susceptibility of various diseases such as asthma and rheumatoid arthritis (RA) (16). An increased risk of frailty due to shorter leukocyte telomere length was also confirmed by MR analysis (17). However, the causal association between circulating immune cell counts and frailty remains under-explored. To address this research gap, we employed the MR approach using recently published genome-wide association studies (GWAS) data on peripheral immune cell phenotypes, as well as the FI (18), and further performed two-step MR to investigate the mediating pathways between immune cell and frailty.

## 2 Materials and methods

### 2.1 Study design

**Figure 1:**
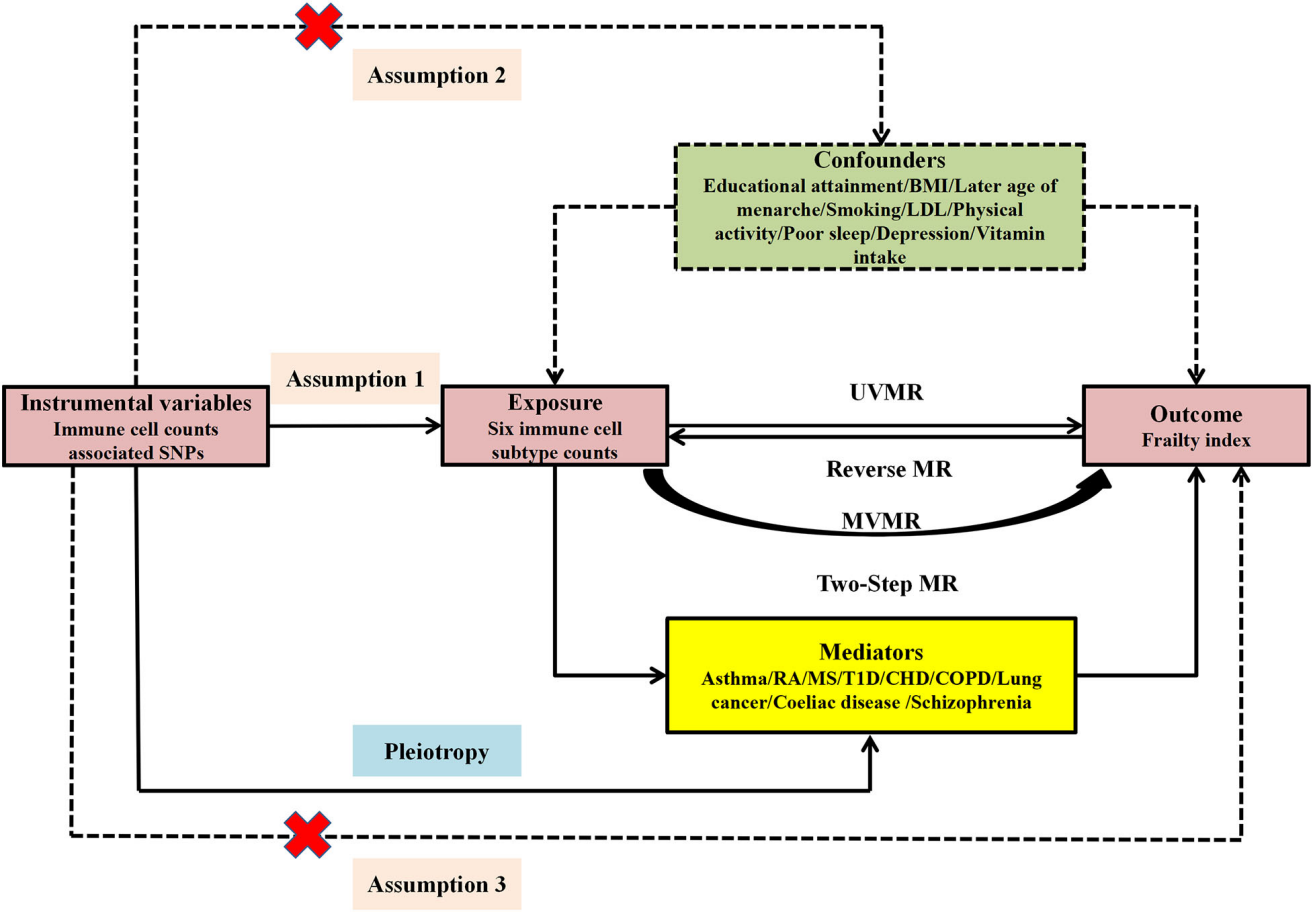
Study design and three corresponding assumptions of this MR study. SNPs, single nucleotide polymorphism; UVMR, univariate MR; MVMR, multivariate MR; BMI, body mass index; LDL, low-density lipoprotein cholesterol; RA, rheumatoid arthritis; MS, multiple sclerosis; T1D, Type 1 diabetes; CHD, coronary heart disease; COPD, Chronic obstructive pulmonary disease.

Single-nucleotide polymorphisms (SNPs) served as instrumental variables (IVs) in the MR investigation. To ensure reliable results, three critical assumptions pertaining to the IVs were addressed: Assumption-1, IVs must exhibit a robust correlation with immune cell subtype count; Assumption-2, IVs should be independent of any potential confounders; Assumption-3, IVs do not influence frailty independently of immune cell subtype counts (**Figure 1**).

This study employed univariate MR (UVMR) and reverse MR to investigate the bidirectional correlation between immune cell counts and FI. Owing to the interplay among the six immune cell subtypes, multivariate MR (MVMR) was conducted to independently ascertain the impact of each cell type on FI. Ultimately, targeting immune cell subtype count with positive causal links to FI in MVMR result, the two-step MR analysis was utilized to explore the mediators in the causal correlation linking immune cell subtype counts and FI (**Figure 1**).

### 2.2 Data sources

The six leukocyte traits investigated were leukocytes, neutrophils, monocytes, lymphocytes, eosinophils, and basophils. The Blood Cell Consortium’s recent extensive meta-analysis of European ancestry (N=563,946) provided the GWAS data for six leukocyte traits. Corrections were made for variables such as age, gender, age-squared, the initial 10 principal components, and cohort-relevant covariates in this GWAS study (19). The Integrative Epidemiology Unit (IEU) OpenGWAS database allows retrieval of exposure data pertaining to the identifier “ieu -b-29 to -33” (**Supplementary Table S1**).

Summary data of frailty was gathered from a recent GWAS meta-analysis comprising European-descended individuals (N = 175,226) from UK Biobank and Swedish TwinGene cohorts (18). In this GWAS research, frailty was gauged using the FI as measure, which relied on the accumulation of 44–49 self-reported statuses concerned with the symptoms, disabilities, and diagnosed diseases that were accumulated throughout an individual’s lifespan (**Supplementary Table S1**).

Previous MR research indicated a causal relationship between immune cell subtype count and several diseases, including asthma, RA, multiple sclerosis (MS), type 1 diabetes (T1D), coronary heart disease (CHD), chronic obstructive pulmonary disease(COPD), lung cancer, coeliac disease, and schizophrenia (16, 20, 21). In this MR study, these possible mediators were included in the two-step MR for mediation analysis. The GWAS data for mediating factors were obtained from the IEU OpenGWAS database. Data sources for these mediators were detailed in **Supplementary Table S1**.

### 2.3 Selection of instrumental variables

In constructing the IVs, genetic variants identified as index SNPs significantly associated (P <5E−08) with leukocyte and subtype counts were selected. To ensure independence among the IVs, variants exhibiting potential linkage disequilibrium (r^2^<0.001 within 10 Mb) were removed. Additionally, SNPs showing significant association with FI (*P <5E−05*) were pruned to improve the validity of our instrumental variables. To harmonize the IVs, SNPs that fail to match or exhibit minor allele frequencies (MAF) less than 0.3 were discarded. We eliminated SNPs associated with outcomes and confounders to prevent possible pleiotropic effects by the PhenoScannerV2 database (http://www.phenoscanner.medschl.cam.ac.uk/) (**Supplementary Table S2**). SNPs classified as weak instrument variables due to F-statistics below 10 were subsequently removed.

Additionally, we assessed the instrument strength for the MR pairs by evaluating the F statistic values [F = ((R^2^/(1 − R^2^)) × ((N − K − 1)/K)]. Concretely, N represents the sample size of the exposure data and R^2^ represents the explained variance of the genetic instruments. SNPs that were identified as weak instrument variables, indicated by F-statistics below 10, were subsequently excluded.

### 2.4 Statistical analysis

The inverse variance weighted (IVW) served as the principal MR analysis method, and IVW with multiplicative random effects method provides a concise estimation and takes into account potential heterogeneity. Consequently, in the presence of heterogeneity, the random-effects IVW models was implemented; otherwise, the fixed-effect IVW model was utilized. To strengthen the robustness of the study outcome, a series of sensitivity analyses were performed, encompassing MR Egger, weighted median, and weighted model.

Pleiotropy was evaluated using the MR Egger and MR pleiotropy residual sum and outlier (MR-PRESSO) approaches. Outliers identified by MR-PRESSO approach were removed, and the IVW results of reassessed MR causality will be showed as MR-PRESSO-IVW. The Cochran’s Q test IVW approach and I^2^ test were employed for heterogeneity analysis, along with a “leave-one-out” sensitivity analysis, which revealed that the causative impact of exposure on the outcome was not governed by single SNPs. To mitigate the risk of type I errors in multiple comparisons, results of UVMR, reverse MR, MVMR and mediation analysis were adjusted using the Bonferroni method, with P < 0.05/n exposures signifying statistical significance.

For MVMR analysis, IVW was the dominant analytical approach employed, with MR-Egger, MR-Lasso, and MR-Median utilized for sensitivity analyses. Two-step MR analysis was implemented to gauge the mediating influence. In the first step, IVs for immune cell subtype count were deployed to access the causal effect of exposure on the mediators. In the second step, IVs for the mediators were adopted to investigate the causal effect of mediators on FI.

The analyses were executed utilizing the Two-Sample MR, MVMR, MR-PRESSO, and MR- intermediary packages within R v.4.2.3 (www.r-project.org), and power computations were accomplished with the aid of an online instrument. (https://shiny.cnsgenomics.com/mRnd/).To monitor bias due to sample overlap in exposure and outcomes, an online tool (https://sb452.shinyapps.io/overlap/) was used to assess the incidence of type 1 errors.

## 3 Results

### 3.1 UVMR analysis of the causal relationship between immune cell counts and FI

**Figure 2:**
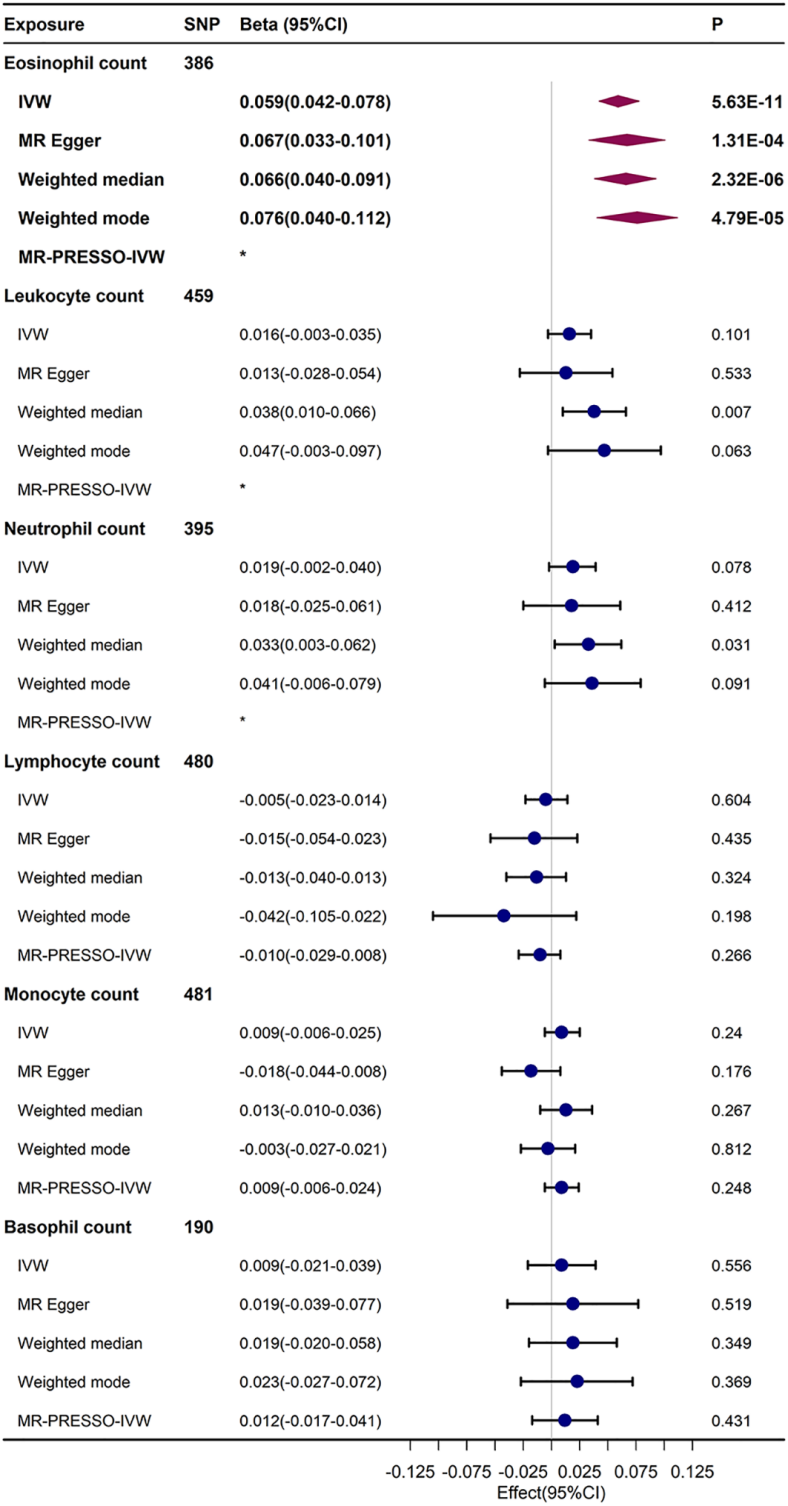
UVMR results regarding the relationship between immune cell counts and FI. The MR-PRESSO-IVW and IVW results of re-MR analysis after the removal of outliers that were probed by MR-PRESSO analysis.*No outliers were detected in the MR-PRESSO analysis.

In standard IVW UVMR analysis, after eliminating pleiotropic SNPs, there was evidence of genetically predicted increased eosinophil count prompting an elevation in FI (β = 0.059, 95% confidence interval [CI], 0.042–0.078, *P* = 5.63E-11). The estimates also aligned with the sensitivity analysis outcomes of MR–Egger, weighted median, and weighted mode (**Figure 2**). The MR-PRESSO analysis results did not indicate the presence of any bias due to outliers in the MR analysis of eosinophil count and FI (**Supplementary Table S3**). After Bonferroni correction(*P*<0.05/6), the causal relationship between eosinophil count and FI remained statistically significant. Because part of the data in both exposures (456,789) and FI (164,610) were from the UK Biobank, there was a possibility of sample overlap. When the maximum sample overlap rate was considered, the online bias calculation results showed that the incidence of type 1 error was 0.05 (**Supplementary Table S4**).

The UVMR results showed a trend towards an increased FI in total leukocyte count (β = 0.016, 95% CI, –0.003–0.0035), neutrophil count (β = 0.019, 95% CI, 0.019–0.04), and basophil count (β = 0.009, 95% CI, –0.021–0.039), but this trend was not statistically significant in the IVW and sensitivity analyses. The findings from the IVW and sensitivity analyses indicated a potential trend of decreasing FI with an increase in lymphocyte count (β = –0.005, 95% CI, –0.023–0.014), but this was not statistically meaningful. However, the causal relationship between monocyte count and FI in the UVMR analysis was not stable and not statistically significant.

**Figure 3:**
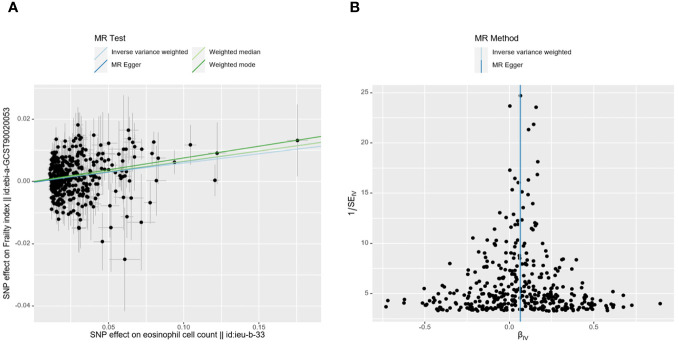
MR plots for reviewing relationship between eosinophil count and FI using UVMR. **(A)** scatter plot of SNP effects on eosinophil count *vs*. FI, with the slope of each line corresponding to the estimated MR effect per method. The data are expressed as raw β values. **(B)** funnel plot to assess heterogeneity; a smaller spread and symmetric distribution indicate lower heterogeneity with no horizontal pleiotropy.

Ultimately, the Egger-intercept *P*-value was not statistically significant for any of the considered outcomes, indicating absence of horizontal pleiotropy (**Supplementary Table S5**). **Supplementary Tables S5**, **6** displayed the results of heterogeneity analysis and power calculations. Moreover, the leave-one-out analysis results suggested that the causal association between eosinophil count and FI remained stable and was not affected by the single SNP (**Supplementary Table S7**). The scatter and funnel plots of the UVMR tests for eosinophil count also showed a consistent trend (**Figure 3**).

### 3.2 Reverse MR analysis of the causal relationship between FI and immune cell counts

The reverse MR approach was utilized in this study, with FI investigated as the exposure and limmune cell counts examined as outcomes. In reverse MR, the IVW results showed causal relationships between the FI and eosinophil count (β = 0.116, 95% CI, 0.003 – 0.230, *P* = 0.044) and lymphocyte count (β = 0.092, 95% CI, 0.011–0.174, *P* = 0.026); however, based on the MR–Egger results, these causal relationships were denoted in the opposite direction and were not statistically significant. When FI was considered as the exposure, the IVW results of the MR analysis suggested a trend for increased FI to be associated with higher counts of leukocytes (β= 0.033, 95% CI,-0.089–0.156), neutrophils (β = 0.043, 95% CI, -0.051–0.138), and basophils (β = 0.045, 95% CI, -0.017–0.108). However, this trend did not reach statistical significance (all P>0.05). Additionally, while the IVW results indicated a non significant positive correlation between FI and monocyte count (β = 0.015, 95% CI, -0.084– 0.113, P = 0.773), this was in contrast to the MR-Egger results where the direction of the effect was reversed(**Supplementary Table S8**). The results of heterogeneity and pleiotropy test are presented in **Supplementary Table S9**.

### 3.3 MVMR analysis of the causal relationship between immune cell counts and FI

**Figure 4:**
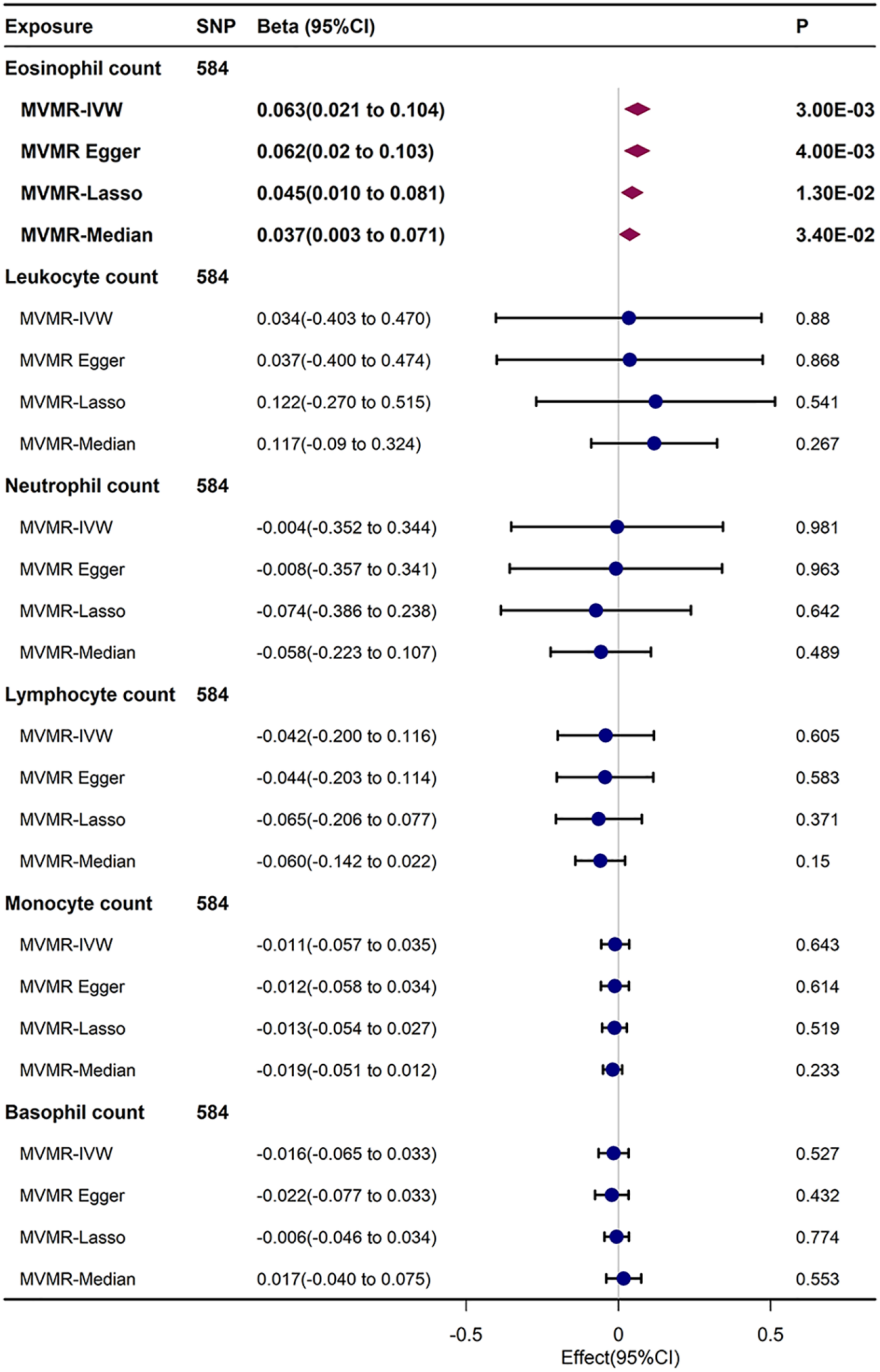
MVMR analysis estimating effect of eosinophil count on FI conditioning on other immune cell subtypes.

In the IVW results of MVMR, upon inspecting the link of genetic liabilities with immune cell counts and FI, eosinophil count retained positive relationship with FI (β= 0.063, 95% CI, 0.021–0.104, *P* = 0.003). This result was generally analogous to those detected in UVMR, MVMR–Egger, MVMR–Lasso, and MVMR-median analyses. After Bonferroni correction (*P* < 0.05/6), the causal relationship between circulating eosinophil count and FI remained statistically significant (**Figure 4**).

### 3.4 Mediation analysis of the causal relationship between eosinophil count and FI

The MVMR results of this study demonstrated independent causal association between circulating eosinophil count and FI. Based on possible mediating factors confirmed in previous MR study (16, 20, 21), we employed a two-step MR analysis. Ultimately, we calculated the indirect effect of eosinophil count on FI via mediators and identified that the mediation effect of asthma amounted to 0.019 (95% CI, 0.013–0.025, *P* = 35.84E-10) with a mediated proportion of 31.732%, while the mediation effect of RA amounted to 0.004 (95% CI, 0.001–0.006, *P* = 1.75E-03) consisting of a mediated segment of 6.411% (**Table 1**). After Bonferroni correction (*P* < 0.05/9), the mediating effects of asthma and RA remained significant.

**Table 1:** The mediating effect of circulating eosinophil count on FI via mediators.

Mediators	Total effect^a^	Direct effect A^b^	Direct effect B^c^	Mediation effect	P	Mediated proportion
	β (95% CI)	β (95% CI)	β (95% CI)	β (95% CI)		(%)
Asthma	0.059 (0.042-0.077)	0.242 (0.194-0.291)	0.078 (0.059-0.097)	0.019 (0.013-0.025)	5.84E-10	31.732
RA	0.059 (0.042-0.077)	0.180 (0.096-0.264)	0.021 (0.012-0.030)	0.004 (0.001-0.006)	1.75E-03	6.411

**^a^**.Causal effect of eosinophil count on FI.

**^b.^** Causal effect of eosinophil count on mediators.

**^c^**.Causal effect of mediators on FI.

RA, rheumatoid arthritis.

## 4 Discussion

This study demonstrated a unidirectional causal effect of circulating eosinophil count on FI, indicating that an elevated eosinophil count resulted in a raised FI. No causal relationships were identified between other leukocyte subtype counts and FI. Importantly, this causal effect was still robust when sensitivity analysis and sample overlap bias were taken into account, and was independent of count of the remaining five leukocyte subtype counts. Then the mediation analysis indicated the causal effect between eosinophil count and FI was mediated in part by asthma and RA.

Our MR results are important as they provide genetically predicted evidence for causal relationship between circulating eosinophil count and F-defined frailty. This study highlighted the potential of eosinophil count serving as novel biomarker for evaluating frailty risk. Of note, even though other immune cell subtypes appeared to be associated with frailty in previous studies (8–13, 22–25), we did not identify evidence of their causal associations on frailty in this MR analysis. Several previous observational studies of the correlation between eosinophil and FI showed no significant differences (10, 11, 25), which contradicted our findings. And some FI studies did not even include eosinophilic counts at baseline (9, 12, 13). Prior observational study results may be affected by sample size, reverse causality and unknown confounding factors, and MR analysis enabled to avoid these biases and provided evidence for causality. Reverse MR revealed that alterations of FI did not precipitate changes in any immune cell subtypes counts, indicating that the causal effect of eosinophil counts on FI was unidirectional and irreversible. Taking into account the potential influence of other immune cell subtype counts, our findings using MVMR method indicated the independent causal effect of eosinophil on FI was not confounded by other immune cell subtypes.

The mechanism by which circulating higher eosinophil count may cause frailty remains unclear, but it is widely accepted that frailty is associated with inflammation and heightened inflammatory responses. Despite comprising less than 5% of circulating leukocytes, eosinophils play a significant role in inflammatory pathways. Eosinophils can secrete an array of granule proteins that display significant cytotoxic effects to promote inflammation (26). Moreover, eosinophil granules not only encapsulate cytokines, chemokines, growth factors, and lipid mediators but also selectively unleash them following diverse stimuli, participating in the progression of inflammation, and the excessive activation of eosinophils can cause exacerbation of inflammation and tissue damage (26, 27). Elevated eosinophil levels in blood or tissue may lead to the development of hypereosinophilic syndrome (HES), leading to pulmonary, cutaneous, gastrointestinal, and cardiac disorders (28). Patients with HES typically present with symptoms such as fatigue, myalgia, weight loss, and fever, which are also commonly observed in frailty patients. Recent MR research suggested causal association between eosinophil and a spectrum of diseases (16, 20, 21). Given that the FI score was based on the accumulation of 44-49 self-reported statuses, which include several diagnosed diseases, we employed mediation analysis to investigate potential shared disease pathways influencing this causal link and confirmed that both asthma and rheumatoid arthritis acted as partial mediators.

This MR study demonstrated that elevated eosinophil count may increase FI scores by raising the risk of asthma. Eosinophils play a pivotal role in immune dysfunction associated with asthma. An investigation involving 130 000 asthma patients in the United Kingdom revealed that an elevated peripheral eosinophil count was linked to a heightened risk of exacerbating asthma and decreased treatment potency (29). A randomized controlled trial confirmed that compared to traditional treatment approaches, a therapeutic strategy aimed at eosinophilic inflammation yielded better asthma outcomes (30). A study involving half a million individuals in the UK revealed that after adjusting for demographic and social–behavioral factors, asthma increased the risk of frailty and pre-frailty among elderly individuals (31). Another 26-year longitudinal study of French adults confirmed a similar conclusion (32). Further exploration is needed to comprehend the mechanisms causing the relationship between asthma and frailty. Several of the inflammatory biomarkers observed in individuals with frailty were also detected in individuals with asthma (33, 34), suggesting that asthma and frailty may share common inflammatory pathways. The two-step MR results in this study also provide the first evidence of causal relationship for genetically predicted asthma and increased risk of frailty. Exploring the potential of therapies targeting eosinophilic inflammation to mitigate asthma-induced frailty may represent a promising novel therapeutic approach.

Our study demonstrated that RA served as a mediator in the process of increased eosinophil count-induced frailty, which was consistent with existing MR results (16). RA is a systemic autoimmune disorder defined by chronic inflammation occurring within synovial joints, the main symptoms of which are restricted joint movement and deformity. Eosinophils have been confirmed to play roles in RA-associated inflammation, which is characterized by heightened serum eosinophil cationic protein levels and peripheral eosinophil count elevation at baseline and served as prognostic indicators for adverse outcomes in early-stage RA (35). Patients with RA suffering from frailty exhibited symptoms that include sarcopenia, fatigue, and low activity. The prevalence of frailty and pre-frailty in RA patients was comparable to or greater than in older adults cohorts (36). The present study also confirmed for the first time the causal relationship between genetically predicted RA and increased frailty risk.

In clinical practice, an incidental increase in eosinophil count during routine blood count analyses often received insufficient attention. This MR study underscored the significance of eosinophils in frailty, offering a new avenue for future research, especially in further elucidating the nuanced biological roles of eosinophils in frailty progression. The discovery that gene-predictive elevated eosinophil counts may increase FI scores by raising the risk of asthma and RA, presented a new perspective in understanding the biological underpinnings of frailty. Incorporating eosinophil count monitoring into standard clinical routines could aid in identifying vulnerable patients with heightened frailty risk and providing targeted preventative and therapeutic measures.

Based on our knowledge, this marks the first investigation elaborating causal connection between eosinophils and frailty. By employing bidirectional UVMR and MVMR methods, we confirmed the directionality and independence of this causal relationship and explored mediating factors. The findings provide a more comprehensive view of this causal pathway. This study’s precision was affirmed by the utilization of a sizable bulk of GWAS data from the European population, while rigorous screening of IVs and various sensitivity analyses have guaranteed the stability of the study results. The limitations of this research should be acknowledged as well. Validation of the eosinophil count–frailty relationship across various frailty evaluation methods is crucial to avoid confounding factors. And due to the intricate mechanism linking eosinophil count and frailty, other mediating factors or inflammatory and immune mechanisms via causal pathways need to be further explored. Furthermore, the magnitudes and proportions of the mediating effects of asthma and RA within this causal pathways may be influenced by the prevalence of these diseases in this FI datasets utilized, and more research validation is necessary. Additionally, our study results pertain primarily to European populations, and generalization to other populations warrants further investigation. Most critically, our findings necessitate subsequent verification in real-world study and wet lab experimentation. Further research is also essential to explore the associations between more refined immune cell subtypes and FI, such as B cell, T cell, and monocyte subsets.

## 5 Conclusions

This MR study provided potent genetic proof for causal association between genetically predicted increased circulating eosinophil count and higher frailty risk in European ancestry, and asthma and RA mediated part of this effect. Additional research is warranted to validate these findings, and in-depth understanding of relevant mechanisms may promote the innovation of frailty prevention strategies.

## Data availability statement

The original contributions presented in the study are included in the article/**Supplementary Material**, further inquiries can be directed to the corresponding author/s.

## Ethics statement

Ethical approval was not required for the study involving humans in accordance with the local legislation and institutional requirements. Written informed consent to participate in this study was not required from the participants or the participants’ legal guardians/next of kin in accordance with the national legislation and the institutional requirements.

## Author contributions

XG: Conceptualization, Investigation, Methodology, Visualization, Writing – original draft. YZ: Conceptualization, Investigation, Methodology, Resources, Writing – original draft. YL: Data curation, Investigation, Software, Writing – review & editing. JL: Data curation, Visualization, Writing – review & editing. JZ: Supervision, Writing – review & editing. HL: Supervision, Writing – review & editing. CC: Conceptualization, Funding acquisition, Writing – review & editing. JY: Conceptualization, Funding acquisition, Writing – review & editing.
